# The Impact of Progesterone Administration Routes on Endometrial Receptivity and Clinical Outcomes in Assisted Reproductive Technology Cycles

**DOI:** 10.7759/cureus.62571

**Published:** 2024-06-17

**Authors:** Hiren Gajjar, Jwal Banker, Shiva Murarka, Parth Shah, Nidhi Shah, Lakshmi Bhaskaran

**Affiliations:** 1 Reproductive Genetics, Neuberg Center for Genomic Medicine, Ahmedabad, IND; 2 Obstetrics and Gynecology, Nova Pulse IVF Center, Ahmedabad, IND; 3 Hematology and Oncology, Dartmouth-Hitchcock Medical Center, Lebanon, USA; 4 Genetics, Dartmouth-Hitchcock Medical Center, Lebanon, USA; 5 Biotechnology and Microbiology, Kadi Sarva Vishwavidyalaya, Gandhinagar, IND

**Keywords:** endometrial receptivity analysis, endometrial receptivity, in vitro fertilization, infertility, progesterone administration

## Abstract

Introduction

Assisted reproductive technologies (ART) rely on endometrial receptivity (ER) for successful embryo implantation. This study aimed to compare the impact of different progesterone administration routes on ER assessed using optimal time for endometrial receptivity analysis (OpERA) and clinical outcomes in ART cycles.

Methods

A retrospective cohort analysis was conducted on 281 infertile women who underwent in vitro fertilization (IVF). Patients were stratified based on progesterone administration routes: oral and vaginal progesterone (Group 1) vs. intramuscular progesterone (Group 2). OpERA was performed on 257 patients to assess ER. Clinical outcomes, including biochemical pregnancy rate (BPR), clinical pregnancy rate (CPR), implantation rate (IR), and abortion rate (AR), were compared between the groups.

Results

OpERA results showed no significant differences between Group 1 and Group 2 in receptive (51.2% vs. 52.0%, p = 0.857), pre-receptive (44.1% vs. 44.6%, p = 0.933), or post-receptive (4.7% vs. 3.1%, p = 0.496) states. Clinical outcomes, including BPR (59.9% vs. 60.9%, p = 0.903), CPR (50.0% vs. 56.5%, p = 0.463), IR (52.5% vs. 55.3%, p = 0.748), and AR (44.3% vs. 45.6%, p = 0.882), did not significantly differ between the groups.

Conclusion

Progesterone administration routes did not significantly affect ER or clinical outcomes, highlighting the need to prioritize understanding and enhancing ER instead of solely focusing on progesterone delivery methods. Identifying molecular pathways or biomarkers could improve receptivity and optimize ART, ultimately improving pregnancy outcomes.

## Introduction

Infertility poses a significant challenge for many individuals and couples seeking successful pregnancy. Assisted reproductive technology (ART), including in vitro fertilization (IVF), offers hope and continues to evolve to improve conception rates. IVF has become a key approach in ART, providing new opportunities for those experiencing pregnancy difficulties [1]. Recent research has emphasized the importance of endometrial receptivity (ER) along with embryo quality for successful outcomes in IVF [2].

Successful pregnancy outcomes rely on precise coordination between the embryonic stage and ER [3]. The implantation window, or 'window of implantation' (WOI), is critical for reproduction and necessitates precise synchronization with progesterone, which prepares the endometrium for embryo implantation [4]. In complex infertility cases, supplemental progesterone is essential, especially when there are deficiencies in the luteal phase or in natural corpus luteum function [5]. In IVF protocols, selecting the appropriate method of progesterone supplementation and its timing in relation to the WOI are crucial considerations [6]. Currently, progesterone can be administered orally, intramuscularly, or vaginally, with distinct advantages and disadvantages in supporting the luteal phase [7].

Despite its ease of use, oral progesterone supplementation is often ineffective in increasing endometrial secretion owing to poor absorption caused by the hepatic first-pass effect [8]. Progesterone is widely administered intramuscularly during frozen embryo transfer (FET) cycles to optimize serum progesterone levels; however, this approach is not without limitations, including discomfort at the injection site and inflammation [9]. Vaginal progesterone administration, a popular luteal phase support option, provides localized delivery and immediate endometrial regulation; however, allergic reactions and discharge have been reported [10]. Nevertheless, it remains uncertain whether the primary issue lies in progesterone levels for each individual type or if it pertains specifically to the endometrial environment regarding receptivity.

Reproductive medicine has made significant strides, leading to the assessment of ER by using molecular techniques [11]. Transcriptomic and artificial intelligence algorithms can accurately determine the WOI, enabling precise identification of the optimal timing for ER-based embryo transfer (ET) and progesterone administration in subsequent cycles [12]. In India, reproductive medicine has been revolutionized by a molecular ER assessment tool that provides tailored recommendations for ET timing based on the optimal time for endometrial receptivity analysis (OpERA) [13]. However, no research has yet confirmed the effectiveness of this method in improving pregnancy outcomes through the examination of different progesterone administration methods in India.

This study explored the impact of different progesterone administration routes on ER. It emphasizes the significance of understanding these factors in relation to OpERA test results and compares the effectiveness of the oral, intramuscular, and vaginal methods. Additionally, this study investigated the association between these findings and pregnancy outcomes, including biochemical pregnancy rate (BPR), clinical pregnancy rate (CPR), implantation rate (IR), and abortion rate (AR) offering valuable insights to enhance progesterone supplementation strategies.

## Materials and methods

Study design

**Figure 1:**
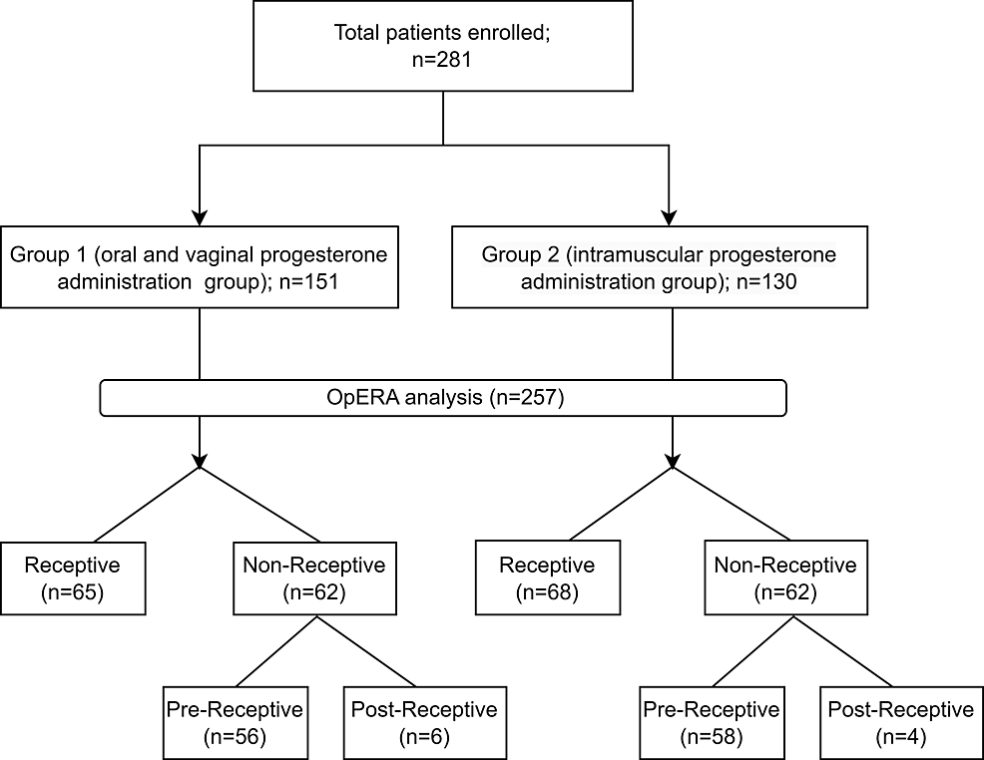
Diagrammatic study design OpERA: optimal timing for endometrial receptivity analysis

This study employed a retrospective cohort design to investigate the impact of distinct progesterone administration routes on the ER and embryo implantation success rates during IVF cycles. Clinical data were collected from electronic medical records of patients who underwent IVF treatment at different IVF centers between September 2022 and November 2023. Approval was obtained from the Institutional Ethics Committee of Gujarat University (Approval Number: GU-IEC (NIV)/02/Proj/013; Date of Approval: June 23, 2022). This study adhered to the ethical guidelines of the 1964 Declaration of Helsinki and its later revisions for all procedures involving human participants. Written informed consent was obtained from all participants. The study design flowchart is shown in Figure 1.

Participants

This study examined 281 female patients who were undergoing thorough evaluations for IVF. These patients did not have any identifiable reasons for multiple failed ET cycles. The study assessed various factors, such as male factor infertility, combined male and female factor infertility, and unexplained infertility. The participants in the study were between the ages of 21 and 45 and had a normal uterus, which was confirmed using 2D/3D ultrasound and/or hysteroscopy. Patients with hydrosalpinx, intramural fibroids >4 cm, stage 3 or 4 endometriosis, and submucosal fibroids or polyps were excluded. The patients were stratified into two groups based on the progesterone administration route.

Preparation and collection of the endometrium and progesterone administration method

Every patient received hormone replacement therapy (HRT) according to a standardized FET protocol. A transvaginal ultrasound was conducted on the second day of the menstrual cycle to eliminate the possibility of cysts or abnormalities. The process of preparing the endometrium began on the second day, which included giving the patient 8 mg of estradiol hemihydrate in two separate doses. Successive ultrasonography examinations were performed until the thickness of the endometrium reached a threshold greater than 7 mm. Continued delivery of estradiol was maintained, and two distinct progesterone medications were administered for a duration of five days, following the blastocyst transfer technique. Group 1 received oral progesterone combined with vaginal progesterone supplementation, whereas Group 2 received intramuscular progesterone medication. The distribution of patients into these groups was determined based on the clinician's pre-established treatment plans, considering factors such as patient history, clinical indications, and previous responses to progesterone treatments. The dosage, formulation, concentration, frequency, and duration of progesterone supplementation were standardized and documented for each group. Following a period of five days, a procedure known as endometrial sampling was conducted using a pipelle. This allowed for the collection and storage of endometrial tissue, which was then used for OpERA testing.

Evaluations of OpERA and suggestions for the WOI

The collected endometrial samples were shipped to the Neuberg Center for Genomic Medicine (NCGM) for analysis using the OpERA test. This test involves the extraction of RNA from endometrial samples, followed by conversion of RNA into complementary DNA (cDNA) through reverse transcription. Subsequently, the cDNA was amplified and sequenced using an advanced Illumina Novaseq 6000 platform (Illumina, Inc., San Diego, USA). RNA expression levels were assessed using fragments per kilobase of transcripts per million mapped reads. ER was evaluated using a machine-learning model based on the predicted gene assortment from the NCGM. The OpERA test provided outcomes indicating the endometrium's receptive (R) or nonreceptive (NR) states. The NR results were further classified as pre- or post-receptive, suggesting a potential shift in WOI. Based on these OpERA results, embryos were transferred during the optimal WOI. In cases where the OpERA results indicate a pre-receptive phase, it is recommended to delay the transfer; conversely, if the OpERA results indicate a post-receptive phase, the transfer timing should be advanced [13]. This means that if the endometrium is post-receptive, the current cycle should be canceled, and the ET should be scheduled earlier in the next cycle to coincide with the optimal WOI.

Clinical outcome parameters

The primary outcome measures included ER status based on OpERA test results, BPR, CPR, IR, and AR in the two distinct progesterone administration groups. BPR indicates the percentage of pregnancies confirmed only by biochemical evidence, such as a positive pregnancy test, but without the detection of a gestational sac or fetal heartbeat. CPR represents the percentage of ETs that result in the detection of a gestational sac or a definitive clinical pregnancy. IR measures the percentage of embryos that were successfully implanted into the endometrium. AR reflects the percentage of clinical pregnancies ending in spontaneous abortion or miscarriage before viability.

Statistical analysis

With a sample size of 281, a statistical power analysis led the study's goal of achieving at least equivalency. This allowed for a strong detection of noteworthy differences or equivalency among the groups, with a margin of error of 5% and an 80% confidence level. Statistical analysis involved summarizing continuous variables, such as age, body mass index, endometrial thickness, and the total number of embryos transferred, using descriptive statistics presented as mean values with standard deviation (SD). Categorical variables, including OpERA outcomes, BPR, CPR, and AR, are summarized as percentages. To compare continuous variables between groups receiving different progesterone administrations, an independent sample t-test was employed. Statistical significance was determined at a threshold of P < 0.05, and all analyses were conducted using GraphPad version 8.0 (GraphPad Software, San Diego, USA).

## Results

Patient characteristics

A total of 281 patients were included in the study and divided into two groups based on progesterone administration: Group 1 received oral and vaginal progesterone (n = 151), whereas Group 2 received intramuscular progesterone (n = 130). No statistically significant differences were identified in terms of age (34.5 ± 4.7 years vs. 33.9 ± 4.5 years; p = 0.310), BMI (27.1 ± 5.4 kg/m² vs. 26.9 ± 5.6 kg/m²; p = 0.749), endometrial thickness (8.0 ± 1.2 mm vs. 8.4 ± 1.2 mm; p = 0.193), or total number of embryos transferred (1.9 ± 0.9 vs. 1.7 ± 1.0; p = 0.297) between the two groups (Table 1).

**Table 1:** Patient characteristics for the study

	Group 1 (oral and vaginal progesterone administration method)	Group 2 (intramuscular progesterone administration method)	p-value
No. of patients	151	130	
Age (years, mean ± standard deviation)	34.5 ± 4.7	33.9 ± 4.5	0.31
Body mass index (BMI) (kg/m²)	27.1 ± 5.4	26.9 ± 5.6	0.749
Endometrial thickness (mm)	8.0 ± 1.2	8.4 ± 1.2	0.193
Total number of embryos transferred	1.9 ± 0.9	1.7 ± 1.0	0.297
Reason for infertility			
Male factor	13/151	9/130	
Combined male and female factor	7/151	6/130	
Unexplained	131/151	115/130	

OpERA test results

**Figure 2:**
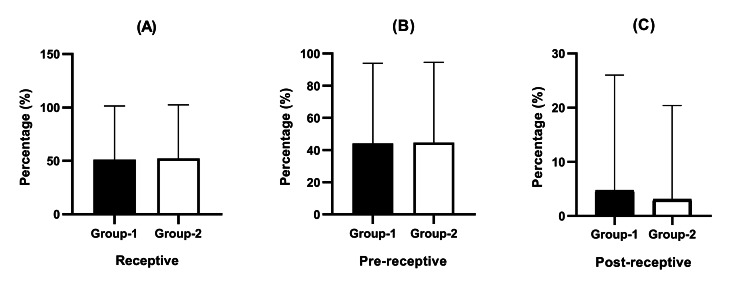
Comparison of OpERA test for receptive, pre-receptive, and post-receptive endometrium in two groups ER outcomes were evaluated in two groups: Group 1 comprised patients receiving combined oral and vaginal progesterone administration, while Group 2 consisted of patients receiving intramuscular progesterone administration. The OpERA analysis compared the distribution of (A) receptive, (B) pre-receptive, and (C) post-receptive endometrium in both groups. Data presented as means ± SD revealed no statistically significant differences in ER between the two progesterone administration routes. ER: endometrial receptivity; SD: standard deviation, OpERA: optimal time for endometrial receptivity analysis

Out of the 281 patients enrolled, 257 individuals participated in the OpERA assessment. Of these, 127 patients were assigned to Group 1, and 130 patients were assigned to Group 2. The remaining patient sample is awaiting the outcome of an OpERA test. The quality of the blastocyst was evaluated using Gardner's scoring system, specifically emphasizing that a blastocyst with a Gardner's score of 4BB or more is considered to be of good quality. Significantly, the quality of blastocysts was found to be similar in the two groups. No statistically significant differences were observed between the two datasets for the OpERA results. Specifically, the percentage of patients displaying receptive OpERA results was similar between Group 1 and Group 2 (51.2% vs. 52.0%, p = 0.857). Additionally, there were no notable differences between the groups in the proportion of pre-receptive (44.1% vs. 44.6%, p = 0.933) or post-receptive (4.7% vs. 3.1%, p = 0.496) patients. The OpERA findings are depicted in Figure 2, which illustrates the distribution of patients across varying receptive states.

Clinical outcomes following OpERA testing

**Figure 3:**
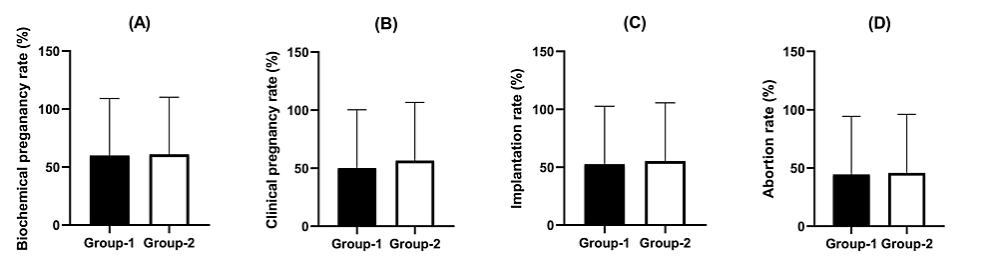
Comparison of clinical outcomes after OpERA analysis by progesterone administration route Clinical results were compared in two progesterone administration route groups after OpERA analysis. Group 1 comprised patients receiving combined oral and vaginal progesterone, while Group 2 consisted of patients receiving intramuscular progesterone. The analysis compared the distribution of biochemical pregnancy rate (A), clinical pregnancy rate (B), implantation rate (C), and abortion rate (D) between the groups. Data presented as means ± SD revealed no statistically significant differences in any clinical outcomes between the two progesterone administration routes. OpERA: optimal time for endometrial receptivity analysis; SD: standard deviation

A total of 163 patients who underwent ET were eligible for evaluation of clinical outcomes based on OpERA analysis. This evaluation included 117 patients assigned to Group 1 and 42 patients assigned to Group 2. Data for the remaining patients are pending as they await definitive clinical outcomes. The percentages of patients who achieved BPR, CPR, IR, and AR after OpERA were compared between the two groups. No significant differences were observed in BPR (59.9% vs. 60.9%; p = 0.903), CPR (50.0% vs. 56.5%; p = 0.463), IR (52.5% vs. 55.3%; p = 0.748), or AR (44.3% vs. 45.6%; p = 0.882) between groups (Figure 3).

## Discussion

The OpERA test provides a unique perspective on ER by enabling exploration of the relationship between intramuscular progesterone and combined oral and vaginal progesterone administration methods. By leveraging gene expression analysis to assess ER, this study offers an objective view of progesterone administration techniques and their impact on fertility outcomes.

This study examined the influence of mid-luteal progesterone administration on ER and embryo implantation to clarify whether it directly enhances implantation or involves immune regulation in pregnancy outcomes. Our findings suggest that mid-luteal progesterone administration alone may not be directly associated with positive pregnancy outcomes, indicating that other biological processes may also contribute to successful implantation and pregnancy.

This study discovered no correlation between transcriptome profiles or endometrial metabolites and intramuscular progesterone levels. However, recent clinical studies have underscored the importance of maintaining a minimum intramuscular progesterone level during HRT cycles for ET to sustain ongoing pregnancies and achieve live birth rates [14]. Notably, patients with intramuscular progesterone levels below 8.8 ng/mL exhibited lower live birth rates and higher clinical miscarriage rates [15]. While progesterone administration is crucial for immune system adjustments, achieving ER may not depend solely on it, as evidenced by the potential for receptivity even with low intramuscular progesterone levels [16].

In addition, combined oral and vaginal progesterone therapy did not significantly affect the outcomes of the OpERA test, highlighting the need to explore alternative strategies for optimizing ER. Previous research has demonstrated significantly elevated vaginal progesterone levels with intravaginal administration compared with intramuscular administration [17]. While intramuscular progesterone increases serum progesterone levels more than vaginal progesterone does, the clinical significance of these differences remains unclear. Recent studies suggest that intramuscular injections may lead to more favorable outcomes, emphasizing the importance of maintaining elevated serum progesterone levels [18,19].

Our findings align with those of a previous study comparing intramuscular and vaginal progesterone supplementation, which demonstrated similar pregnancy outcomes across various ET cycles [20]. This consistency underscores the robustness and reliability of our results, suggesting that the choice of progesterone administration method may not significantly affect pregnancy outcomes. Additionally, our findings are consistent with prior research indicating that physiological differences between fresh and FET cycles do not notably affect the efficacy of progesterone administration methods [21]. This finding suggests that personalized progesterone supplementation strategies maintain their effectiveness across different cycle types, highlighting the importance of tailored treatment approaches in assisted reproduction. However, our results differ from those of other studies that showed improved outcomes with vaginal progesterone gel combined with dydrogesterone [22] or superior results with intramuscular injection [23]. Notably, previous studies lacked molecular-based assessments of ER, which may have influenced their findings. Our study, incorporating gene expression-based analysis, provides an objective measure of receptivity, enhances the reliability of our results, and contributes to the current understanding of ART.

ER involves a complex interplay of numerous molecules, many of which undergo cyclic hormonal regulation during the menstrual cycle [24]. In addition to hormone metabolites present in the endometrium, various coactivators, inflammatory mediators, and other factors contribute to the transmission of progesterone-driven responses [25]. Diminished endometrial responsiveness to progesterone, a factor associated with progesterone resistance [26], may negatively impact ER, potentially leading to implantation failure or suboptimal pregnancy outcomes. Hormonal fluctuations, genetic predispositions, inflammatory mediators, immunological responses, and endometrial microenvironment are all potential variables that can influence ER.

This study has a number of limitations. One aspect of it is its retrospective nature. This implies that the study may have been subject to bias in comparison to prospective clinical trials. In addition, the limited sample size may have impeded the generalizability of our findings to a larger population. Confounding variables, such as reproductive conditions and medication adherence, can influence progesterone metabolism and ER. Furthermore, the study's limited follow-up duration restricted the assessment of long-term outcomes such as live birth rates. However, this study contributes to the current understanding of ART by investigating the relationship between endometrial responsiveness to the OpERA test and various progesterone delivery methods. Additionally, gene expression-based analyses provide an objective measure of receptivity, establishing a robust foundation for evaluating endometrial responsiveness using different progesterone delivery methods.

## Conclusions

In conclusion, our study underscores the complex relationship between the progesterone administration method and ER, as evaluated using the OpERA test. While systemic progesterone levels are crucial for sustaining pregnancy, our findings suggest that, instead of exclusively focusing on progesterone delivery methods, it may be advantageous to prioritize understanding and enhancing ER. Exploring specific molecular pathways or identifying biomarkers associated with the receptive endometrium could provide novel insights for improving receptivity and optimizing ART, ultimately enhancing pregnancy outcomes in clinical practice. Further research is needed to investigate additional factors influencing ER and refine progesterone administration strategies.
